# Optimizing the design of invasive placebo interventions in randomized controlled trials

**DOI:** 10.1002/bjs.11509

**Published:** 2020-03-18

**Authors:** S. Cousins, N. S. Blencowe, C. Tsang, K. Chalmers, A. Mardanpour, A. J. Carr, M. K. Campbell, J. A. Cook, D. J. Beard, J. M. Blazeby

**Affiliations:** ^1^ National Institute for Health Research (NIHR) Biomedical Research Centre at University Hospitals Bristol NHS Foundation Trust and University of Bristol Surgical Innovation Theme; ^2^ Medical Research Council ConDuCT‐II Hub for Trials Methodology Research Bristol Centre for Surgical Research, Population Health Sciences, Bristol Medical School; ^3^ Division of Surgery University Hospitals Bristol NHS Foundation Trust Bristol; ^4^ Nuffield Department of Orthopaedics Rheumatology and Musculoskeletal Sciences, NIHR Biomedical Research Centre, University of Oxford; ^5^ Royal College of Surgeons (England) Surgical Interventional Trials Unit University of Oxford Headington Oxford; ^6^ Health Services Research Unit University of Aberdeen Aberdeen UK

## Abstract

**Background:**

Placebo‐controlled trials play an important role in the evaluation of healthcare interventions. However, they can be challenging to design and deliver for invasive interventions, including surgery. In‐depth understanding of the component parts of the treatment intervention is needed to ascertain what should, and should not, be delivered as part of the placebo. Assessment of risk to patients and strategies to ensure that the placebo effectively mimics the treatment are also required. To date, no guidance exists for the design of invasive placebo interventions. This study aimed to develop a framework to optimize the design and delivery of invasive placebo interventions in RCTs.

**Methods:**

A preliminary framework was developed using published literature to: expand the scope of an existing typology, which facilitates the deconstruction of invasive interventions; and identify placebo optimization strategies. The framework was refined after consultation with key stakeholders in surgical trials, consensus methodology and medical ethics.

**Results:**

The resulting DITTO framework consists of five stages: deconstruct treatment intervention into constituent components and co‐interventions; identify critical surgical element(s); take out the critical element(s); think risk, feasibility and role of placebo in the trial when considering remaining components; and optimize placebo to ensure effective blinding of patients and trial personnel.

**Conclusion:**

DITTO considers invasive placebo composition systematically, accounting for risk, feasibility and placebo optimization. Use of the framework can support the design of high‐quality RCTs, which are needed to underpin delivery of healthcare interventions.

## Graphical abstract

There are challenges in RCTs of invasive interventions with a placebo control. The DITTO framework outlines five stages to optimize invasive placebo design and delivery: deconstruction of the treatment intervention, identification (and removal) of the critical surgical element from the proposed placebo, consideration of risk, feasibility and the role of the placebo in the RCT, and optimization of placebo delivery to ensure effective blinding of patients and trial personnel.

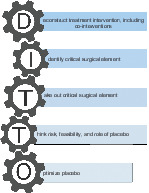

DITTO will be useful

## Abstract (es)

**Antecedentes:**

Los ensayos controlados con placebo juegan un papel importante en la evaluación de las intervenciones sanitarias. Sin embargo, pueden ser difíciles de diseñar e implementar en el caso de intervenciones invasivas, incluida la cirugía. Se necesita un conocimiento profundo de los componentes de la intervención terapéutica (para determinar qué se debe y qué no se debe administrar como parte del placebo). También se precisa de una evaluación del riesgo para los pacientes y de las estrategias para garantizar que el placebo imite el tratamiento de forma efectiva. Hasta la fecha no existen guías para el diseño de intervenciones invasivas con placebo. Este estudio tuvo como objetivo desarrollar un marco para optimizar el diseño y la práctica de intervenciones invasivas con placebo dentro en los ensayos clínicos aleatorizados (ECA).

**Métodos:**

Utilizando la literatura publicada, se desarrolló un marco preliminar para i) ampliar el alcance de los modelos existentes para facilitar la deconstrucción de las actuaciones invasivas, y ii) identificar estrategias para optimizar el placebo. El marco se perfeccionó tras consultar con partes interesadas ​​en ensayos quirúrgicos, metodología de consenso y ética médica.

**Resultados:**

El marco DITTO resultantes consiste en cinco etapas: Etapa 1: deconstrucción de la intervención de tratamiento en sus componentes esenciales y co‐intervenciones; Etapa 2: identificar el(los) elemento(s) quirúrgico(s) básico(s); Etapa 3: eliminar el(los) elemento(s) básico(s); Etapa 4: considerar el riesgo, la viabilidad y el papel del placebo en el ensayo al tener en cuenta los demás componentes; y Etapa 5: optimizar el placebo para garantizar el cegamiento efectivo de los pacientes y del personal del ensayo.

**Conclusión:**

DITTO considera de forma sistemática la naturaleza invasiva del placebo, teniendo en cuenta el riesgo, la viabilidad y la optimización del placebo. El uso de este marco de referencia puede ayudar al diseño de ECAs de alta calidad, necesarios para afianzar la implementación de intervenciones sanitarias.

## Introduction

Randomized controlled trials (RCTs) involving placebo comparators have an important role in the evaluation of healthcare interventions. A key advantage is that they allow estimation of the true effect of the treatment under evaluation by quantifying the placebo response: the effects of concomitant non‐treatment factors, such as setting, patient expectations, interactions with health professionals and the natural course of the disease/condition^1^, ^2^, ^3^. Placebo‐controlled RCTs also provide the methodological ideal in terms of blinding of patients, trial personnel and healthcare professionals to trial group allocation, thereby minimizing the potential for bias. This is important because knowledge of trial group allocation can lead to potentially biased assessments of outcomes, especially for subjective measures such as patient‐reported pain scores^4^, ^5^, and may affect the co‐interventions delivered by clinicians or sought by patients. Although pharmaceutical studies involving placebo drugs are common, placebo‐controlled trials of invasive interventions, including surgery, remain rare.

One of the main challenges arises from ethical concerns about the potential risks arising from exposing patients to placebo interventions requiring incisions (or other forms of access to the body) and the use of anaesthesia. Closely linked is the issue of acceptability of such interventions to clinicians and patients allocated to the placebo group^6^, ^7^, ^8^. Invasive placebo interventions are also challenging practically to design and deliver. It can be difficult to ensure that a placebo intervention is indistinguishable from the treatment, owing to the invasive nature and intrinsic role of surgeons and clinical staff in delivering the intervention. Furthermore, as invasive interventions are complex, comprising multiple interacting components and co‐interventions^9^, it can be difficult to decide which components should and should not be included within the placebo intervention.

A typology^10^ was developed with the aim of facilitating the systematic and comprehensive identification of individual components and steps of invasive treatment interventions. Although this is useful, the design of invasive placebo interventions requires additional considerations, such as identification of the treatment component(s) believed to provide the therapeutic benefit (critical surgical element(s)^11^), minimization of risks, and the selection and monitoring of co‐interventions and placebo optimization strategies to ensure blinding.

Given the complexities inherent in the design and delivery of invasive placebo‐controlled trials, including associated ethical considerations, careful consideration of the design of invasive placebo interventions is important. The aim of this study was to develop a framework to inform the optimal design and delivery of invasive placebo interventions for use in RCTs.

## Methods

Development of the framework was informed by the following steps: expanding the scope of the typology^10^ to facilitate deconstruction of invasive treatment interventions where access to the body is made via incision, natural orifice and percutaneous puncture; identification of placebo optimization strategies from the published literature; review of a preliminary framework by key stakeholders attending an international expert meeting; and refinement of the framework in light of stakeholder feedback.

### Identification of published placebo‐controlled trials of invasive procedures

Articles reporting a protocol or results of RCTs comparing an invasive intervention with placebo, in living humans, identified by a previous systematic review^12^ were included. Articles published between database inception and 31 December 2017 were retrieved from OVID MEDLINE, Embase and the Cochrane Central Register of Controlled Trials (Central) electronic databases. Search concepts related to RCTs, surgery and placebo were used^12^, ^13^. Additional RCTs, with no restriction on publication date, were identified by hand searching references of included articles and expert knowledge. Invasive interventions, where access to the body was gained by incision, percutaneous puncture or endoscopic techniques, were included. In accordance with the review of Wartolowska and colleagues^13^, placebo interventions included surgical placebo, sham surgery, or any intervention intended to mimic the active intervention. Excluded were RCTs evaluating medicinal products (including those delivered before, during and after the invasive procedure) and dental interventions, non‐randomized studies, reviews, editorials, letters or conference abstracts.

Included articles were then reviewed to revise the original typology and identify components of treatment interventions not previously included; and to identify placebo optimization strategies used to ensure blinding of patients and trial personnel to trial group allocation (treatment or placebo)^14^.

### Revising scope of original typology

Section 1 of the original typology, relating to the identification of components delivered within invasive treatment interventions, was updated using methods reported previously^10^. The scope of the typology was widened to permit deconstruction of not only invasive treatment interventions with incisions, but also those where access to the body is gained via a natural orifice or percutaneous puncture^15^. Additionally, the typology was expanded to identify co‐interventions delivered to patients. Co‐interventions were defined as any additional diagnostic or therapeutic interventions delivered to patients as part of the treatment protocol before, during or after the treatment intervention.

Using a deductive approach, the typology was modified iteratively to incorporate any components reported in descriptions of treatment interventions in the published literature not previously included in the typology. The process of iterative modifications consisted of assigning descriptive labels to all reported information about invasive interventions. These labels then informed the addition or modification of components within the typology. Subsets of articles were read and re‐read to understand the data. Where required, existing components of the typology were amended and additional components added. This process was repeated until saturation had been achieved^16^, that is no additional descriptive labels emerged from the articles that required further changes to the typology. The research team met regularly to discuss all findings. The iterative nature of this process allowed the study team to be confident that no descriptions of treatment interventions were emerging from the published studies that required further changes to the typology.

### Identification of placebo optimization strategies

Published literature was reviewed to identify reported strategies for optimizing blinding of patients and trial personnel to trial group allocation (treatment or placebo)^14^. Where placebo optimization strategies identified shared commonalities, they were grouped. For example, strategies aiming to manipulate sensory input were grouped, whether this was through sensory deprivation (such as visual masking) or the delivery of additional sensory cues (for example, verbal cues to simulate treatment components in the placebo group).

### Review of preliminary framework by key stakeholders

A preliminary framework was presented to key stakeholders attending a 2‐day workshop, ‘Methods for Placebo Comparator Group Selection and Use in Surgical Trials’ in December 2018. This workshop was commissioned and jointly funded by the UK Medical Research Council (MRC) and the UK National Institute for Health Research (NIHR), with support from the Bristol Biomedical Research Centre, in response to a commissioned call for a state‐of‐the‐art workshop on this topic. The workshop covered several other aspects of placebo surgery, including definitions, ethics and trial conduct. Attendees were invited based on their national and international expert knowledge in surgical trial methodology, medical ethics and consensus methods, and included surgeons, trialists, funders, ethicists and patient representatives. The preliminary framework was based on: deconstruction of the treatment intervention using the refined typology; identification and omission of the critical surgical element; and use of placebo optimization strategies identified in the literature. This was presented formally during a dedicated session, and attendees were given the opportunity to discuss and provide feedback. Detailed notes of the discussion and comments raised by attendees were taken by dedicated note‐takers.

### Refinement of framework

Detailed notes outlining feedback from key stakeholders were used to update the preliminary framework iteratively within the study team. The final version of the framework was agreed by full detailed discussion within the study team when no further changes were required to address stakeholder feedback and any outstanding methodological requirements for placebo design.

## Results

A total of 96 published placebo‐controlled RCTs of invasive interventions were used to develop the framework^12^. Most were conducted in Europe (40, 42 per cent) and the USA (37, 39 per cent) in gastrointestinal surgery (40, 42 per cent). Approximately two‐thirds randomized fewer than 100 patients (65, 68 per cent) and 31 (32 per cent) involved a single centre.

### Revised typology

The revised typology is shown in *Table* 1.

**Table 1:** Revised typology with potential components of invasive treatment interventions, including co‐interventions

Component	Description
Anaesthesia*	Details of type of anaesthesia delivered to patients, including sedation, local and general anaesthetic
Before access to body (in procedure room)	Events associated with surgical intervention occurring before access to body is gained, e.g. patient positioning, skin preparation, hair removal, dressing in surgical scrub
Access*	Method used to gain access to body. Broadly this can be categorized as incision with cut, through natural orifice or percutaneous puncture
Dissection	Process of exposing an organ, tissue or structure
Irrigation*	Application of any solution across or within an open wound or inside body to achieve wound hydration, remove debris, or assist with visual examination
Resection	Removal of all or part of an organ, tissue or structure
Haemostasis	Stopping of bleeding or arrest of blood circulation in an organ, tissue or structure
Reconstruction	Process of rebuilding, repairing or replacing an organ, tissue or structure. This may include an anastomosis (connection between 2 structures) or insertion of a surgical adjunct such as a mesh or prosthesis
Insertion of surgical adjunct	This relates to insertion of surgical adjuncts not related directly to reconstruction, but inserted during surgical procedure (e.g. drains or feeding tubes)
Intraoperative diagnosis	Further characterization of disease process or anatomy during surgical procedure (e.g. intraoperative cholangiography, blue dye tests or scintigraphy)
Closure/removal of equipment*	Process of closing incision(s) or removing equipment from body
After skin closure	Any event associated with surgical intervention but undertaken after skin closure (e.g. application of dressings or bandages)
Co‐interventions*	Any co‐interventions delivered to patients before, during or after invasive intervention
Other	Any other component not listed above

* Revised typology components.

### Placebo optimization strategies

Seventy‐eight trials (81 per cent) reported at least one placebo optimization strategy (*Table* 2).

**Table 2:** Placebo optimization strategies reported in placebo‐controlled RCTs of invasive interventions

Placebo optimization strategy	No. of RCTs (*n* = 78)	Example – verbatim text from trial article
**Sensory manipulation**	54 (69)	
Visual masking	13 (17)	‘..the blinding of the patient was further ensured by shielding the patients’ view with a vertical drape and aiming the arthroscopy monitors away from the patient's line of vision'^17^
Verbal cues	12 (15)	‘During the sham procedure, physician investigators were required to talk through the procedure steps to facilitate blinding of the patient. Spoken dialogue during the sham procedure mimicked actual use’^18^
Auditory cues	11 (14)	‘Saline was splashed to simulate the sounds of lavage’^19^
Physical cues	9 (12)	‘..the endoscope was manipulated for 30 to 40 minutes to simulate the effect of rotations and manipulations on the esophagus’^20^
Visual cues	5 (6)	‘Several 1 ml syringes will be filled as for the active group and injection will be simulated’^21^
Auditory masking	3 (4)	‘..the patient received over‐the‐ear headphones playing music that ensured auditory isolation and prevented hearing of communication between staff, even before sedation’^22^
Olfactory cues	2 (3)	‘..the methacrylate monomer was opened to simulate the odor associated with mixing of PMMA’^23^
**Use of devices to optimize blinding**	37 (47)	‘The palatal implant insertion tools provided by the manufacturer for the placebo control group did not include the palatal implants, but they were in all other aspects identical to the implant insertion tools used in the treatment group receiving the implant’^24^
**Mimicked timings**	26 (33)	‘The patient was kept in the operation theatre for the amount of time required to perform an actual arthroscopic index shoulder surgery’^25^
**Restricting interaction between blinded and unblinded trial persons**	11 (14)	‘Shortly before each intervention the endoscopist was informed about the patient's group assignment. Subsequent contact between the patient and the endoscopist was minimised’^26^
**Omission of intervention details in trial‐related paperwork**	11 (14)	
Intervention not specified in patient notes	9 (12)	A standardized operation description was written in the patients' charts in order to deprive the nursing staff at the hospital of information on the character of the operation'^27^
Patient billing delayed or withheld	2 (3)	‘Each site will be asked to delay the billing to the subjects in an attempt to keep them blinded as to the procedure they receive. The costs of the vertebroplasty will be billed to the subject's insurance after the one‐month evaluation’^23^
**Unblinded procedurist delivering component of intervention**	3 (4)	‘..the catheter was connected to a lead and passed to an independent technician. The technician then opened a sealed envelope to ascertain the randomization schedule and covertly either connected the catheter to the generator (active IDET group) or did not (sham placebo group). Critically, both surgeon and subject were blinded to this step’^28^

Numbers in parentheses are percentages.

#### *Sensory manipulation*

Sensory manipulation (54 trials, 69 per cent) included the use of cues (auditory, visual, physical and olfactory) to simulate treatment components, and masking (auditory and visual) to prevent patients from receiving sensory cues about which intervention they had received.

#### *Use of devices to optimize blinding*

Thirty‐seven RCTs (47 per cent) described the use of devices to optimize blinding, including the use of ‘deactivated’ devices in the placebo intervention that were disconnected, turned off or incapable of delivering the intervention. In addition, where implants were delivered, there were examples of implant insertion tools created by the manufacturer that did not contain the implant under evaluation, although it was not possible to tell this externally. For example, in an evaluation of palatal implants for snoring and obstructive sleep apnoea, Friedman and colleagues^24^ used palatal implant insertion tools provided by the manufacturer that did not contain the implant, but were in all other aspects identical to those used in the treatment group.

#### *Mimicked timings*

Twenty‐six trials (33 per cent) used mimicked timings, whereby patients receiving the placebo intervention spent the same amount of time in the procedure and/or recovery room as those receiving the treatment.

#### *Restricting interaction between blinded and unblinded trial persons*

In 11 trials (14 per cent), the interaction between trial persons with and without knowledge of trial group allocation was deliberately restricted.

#### *Omission of intervention details from trial‐related paperwork*

In 11 trials (14 per cent), intervention details were omitted from trial‐related paperwork, for example patient notes and hospital bills.

#### *Unblinded procedurist delivering component of intervention*

In three trials (4 per cent), specific components of both treatment and placebo interventions were delivered by an unblinded procedurist, in an effort to blind trial personnel delivering the rest of the intervention.

### Feedback on the preliminary framework from key stakeholders

Two related themes emerged from stakeholder feedback: assessment of potential risks to patients receiving a placebo intervention, such as the need to consider risk related to choice of anaesthesia; and using the role of the placebo intervention in the RCT to guide placebo intervention design. For example, in a trial to identify the mechanism of therapeutic action of the treatment intervention, it would be advantageous to design a placebo intervention that matches the treatment in all components, except the critical element. Conversely, if the purpose of the placebo intervention is primarily to blind trial persons, it may not be necessary to maximize the treatment components delivered in the placebo group. Rather, the use of placebo optimization strategies and the matching of co‐interventions (such as postoperative care) may be used primarily.

### Refinement of framework

**Figure 1:**
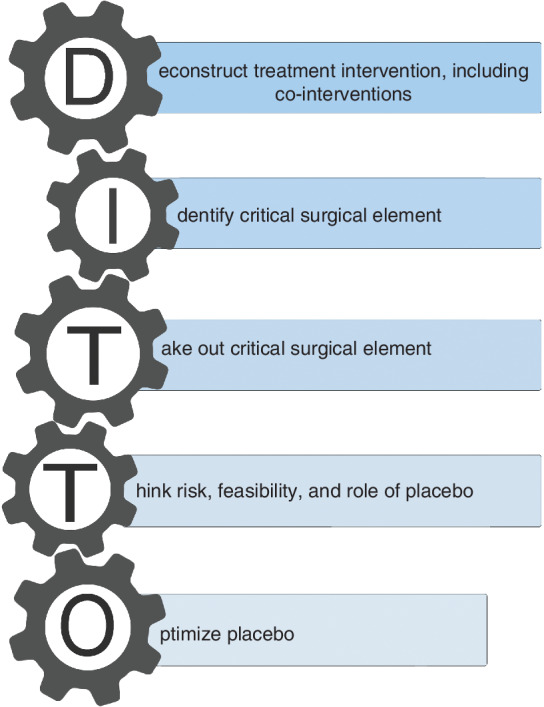
Core stages of the DITTO framework

The preliminary framework was revised following consultation with stakeholders. The resulting DITTO framework consists of five core stages that need to be considered iteratively when designing invasive placebo interventions (*Fig*. 1).

#### *Deconstruct treatment intervention, including co‐interventions*

Here, the updated typology is used to deconstruct the treatment intervention. This results in the production of a comprehensive list of treatment components and steps, including co‐interventions occuring before, during and after the treatment intervention.

#### *Identify critical surgical element*

The critical surgical element(s) is then identified from the comprehensive list of treatment intervention components.

#### *Take out critical surgical element(s)*

The critical element(s) is then removed from the proposed placebo intervention.

#### *Think risk, feasibility and role of placebo in trial*

Once the critical surgical element(s) has been removed, it is important to consider the inclusion of the remaining components/steps, taking account of potential risk to patients, feasibility and the role of the placebo intervention in the RCT (for example, as a control intervention to elucidate the treatment mechanism).

#### *Optimize placebo*

The use of placebo optimization strategies should be considered throughout the design process. For example, if a component of the treatment intervention is deemed to pose an unacceptable degree of potential risk to patients, trialists may instead consider the use of placebo optimization strategies to simulate the delivery of this component.

### Hypothetical application of DITTO framework to design placebo intervention in a trial of appendicectomy *versus* placebo control

*Table* 3 illustrates the use of the DITTO framework in a hypothetical RCT comparing placebo with appendicectomy, which has been suggested to alter the clinical course of ulcerative colitis. The appendicectomy procedure is deconstructed using the updated typology, and the critical surgical element identified and removed. An indication of the potential inclusion/omission of the remaining treatment components is given, as well as placebo optimization strategies that may be used.

**Table 3:** Worked example showing application of DITTO framework to development of a placebo intervention for appendicectomy

Typology components	Treatment intervention (appendicectomy)	Placebo intervention
Anaesthesia	General anaesthetic	General anaesthetic
Before access to body (in operating theatre)	Skin preparation, positioning and draping	Skin preparation, positioning and draping
Access	3 incisions (sizes flexible)	3 incisions (sizes flexible)
	Create pneumoperitoneum and inspect intra‐abdominal organs	Create pneumoperitoneum and inspect intra‐abdominal organs
Dissection	Identify appendix	Identify appendix
	Mobilize appendix and dissect mesoappendix	×
Irrigation	n.a.	n.a.
Resection*	Ligate base of appendix (stapler or endoloop)	×
Remove appendix in bag (optional)	×
Haemostasis	Ligate appendicular artery (using diathermy or clips)	×
Check for bleeding	Check for bleeding
Reconstruction	n.a.	n.a.
Insertion of surgical adjunct	n.a.	n.a.
Intraoperative diagnosis	n.a.	n.a.
Closure/removal of equipment	Closure of fascia of port(s) > 5 mm	Closure of fascia of port(s) > 5 mm
	Subcuticular skin sutures	Subcuticular skin sutures
	Infiltration of local anaesthetic (type and amount flexible)	Infiltration of local anaesthetic (type and amount flexible)
After skin closure	Apply dressings (optional)	Apply dressings (optional)
Co‐interventions	Urinary catheter (optional)	Urinary catheter (optional)
	Antibiotics at time of skin incision	Antibiotics at time of skin incision
	Intraoperative analgesia and fluids	Intraoperative analgesia and fluids
	Postoperative analgesia	Postoperative analgesia
Other	n.a.	n.a.
**Placebo optimization strategies**		
Visual masking	Eye mask while in procedure room and postprocedure recovery room	
Auditory masking	Headphones while in procedure room and postprocedure recovery room	
Mimicked timings	All patients to spend same length of time in operating theatre, recovery rooms and in hospital after procedure	
Restriction of interaction between blinded/unblinded trial persons	Separate healthcare team not present in theatre to look after patients after procedure	
Omission of intervention details in patient notes	Operation notes kept in sealed envelope separate to patient's medical records, only to be accessed in an emergency†	

* Critical surgical elements. n.a., Not applicable; ×, treatment step omitted from placebo appendicectomy.

† With agreed unblinding protocols in place.

## Discussion

Placebo‐controlled trials of invasive interventions can be challenging to conduct, and there is a need to optimize their design and delivery. This study has used published literature and expert opinion to develop the DITTO framework, a standardized methodological framework to optimize the design and delivery of invasive placebo interventions for use in RCTs. The DITTO framework facilitates deconstruction of invasive treatment interventions, and considers systematically which components should, and should not, be delivered within the placebo intervention, considering issues of risk, feasibility and scientific validity. The DITTO framework can be used in the design and delivery of placebo‐controlled trials of invasive interventions, including surgery, to support the generation of the ‘gold standard’ evidence that is needed to underpin delivery of these common healthcare interventions^29^.

Previous work regarding the design of invasive placebo interventions specifically is limited. Pilot and feasibility work conducted before placebo‐controlled trials of invasive interventions has highlighted the importance of assessing patient and clinician acceptability during trial design^6^, ^7^. This includes assessment of the potential risk of delivering components of the treatment intervention in the placebo, including anaesthesia^6^. Assessment of risk and careful trial design is highlighted across publications examining the use of invasive placebo interventions in RCTs^4^, ^30^, ^31^. Risk assessment coupled with consideration of how to optimize scientific validity, by ensuring that the placebo effectively mimics the treatment intervention using placebo optimization strategies while minimizing risk, are key aspects of the DITTO framework. The use of an invasive placebo intervention that fails to blind key trial persons adequately may reflect poor trial design, produce biased results and be difficult to justify ethically. These considerations mirror those important in placebo drug development; not only must the placebo tablet be matched to treatment in terms of visual appearance, size and weight, but assessments of the constituents of the placebo to ensure their inert or inactive status must also be conducted^32^.

A limitation of the DITTO framework in its current form is that does not specify how assessments of risk and feasibility of including components in the placebo intervention may take place. For example, interviews and focus groups with key stakeholders may be used in pilot and feasibility work before the main trial^6^. The use of pilot and feasibility work, which may also provide an opportunity to test placebo optimization strategies^7^ and determine the critical surgical element(s), has not been considered explicitly in the current framework, but is important to consider in future work. Identifying which component(s) provides the critical therapeutic benefit may be difficult, and the degree to which the remaining components contribute to the treatment effect of the intervention requires consideration. In this regard, stakeholder interviews and/or consensus work before the main trial would be important to determine which components should be omitted from the placebo. Furthermore, although the updated typology provides a comprehensive list of components that may be delivered within the treatment intervention, the number and type of steps within any component are likely to vary widely depending on the invasive intervention. Thus, it is not possible to include in the typology individual treatment steps that would be universally applicable^10^. However, expansion of the typology to include a wider array of potential treatment co‐interventions may be possible. Future work to develop the framework should incorporate these issues. Piloting of the framework in RCTs of invasive interventions will facilitate development as well as help assess its usefulness in practice. Fostering links with professional bodies such as the Royal College of Surgeons of England and key stakeholders, including surgeons, funders, journal editors and clinical trials units, will also provide opportunities for further development and implementation of DITTO. Specifically, publication of the framework and presentation at international trials methodology conferences and national Royal College of Surgeons' surgical trials centre meetings will promote development and use of the framework. Future plans to ensure uptake also include an interactive website to facilitate use of DITTO at the point of trial design.

Development work and dissemination may also consolidate the role of the DITTO framework in facilitating the transparent reporting of interventions delivered within placebo‐controlled trials of invasive procedures. Both the TIDieR (template for intervention description and replication)^33^ and SPIRIT (Standard Protocol Items: Recommendations for Interventional Trials)^34^ checklists aim to enhance the transparent reporting of interventions, including surgery. The DITTO framework can facilitate adherence to these guidelines by supporting the deconstruction of the treatment intervention into its constituent components. Furthermore, although the TIDieR and SPIRIT guidelines do not mention invasive placebo interventions specifically, the detailed consideration given to the content of the placebo intervention in DITTO would also promote the transparent reporting of the components and co‐interventions delivered within the placebo. It is expected that future work will develop reporting guidelines specifically for placebo‐controlled trials of invasive procedures.

The DITTO framework provides a formalized, systematic approach to the design and delivery of invasive placebo interventions, which pose specific ethical and practical challenges. Use in future trials of invasive healthcare interventions, including surgery, will support the continued methodological advancements in this area^35^, ^36^. This is key for the generation of high‐quality research data to underpin evidence‐based clinical practice.
